# Dynamic Flow Control over Optical Properties of Liquid Crystal–Quantum Dot Hybrids in Microfluidic Devices

**DOI:** 10.3390/mi14050990

**Published:** 2023-04-30

**Authors:** Artem Bezrukov, Yury Galyametdinov

**Affiliations:** Department of Physical and Colloid Chemistry, Kazan National Research Technological University, 420015 Kazan, Russia; yugal2002@mail.ru

**Keywords:** microfluidics, lab on chip, liquid crystals, quantum dots, confinement, optical response

## Abstract

In this paper, we report developing approaches to tuning the optical behavior of microfluidic devices by infusing smart hybrids of liquid crystal and quantum dots into microchannel confinement. We characterize the optical responses of liquid crystal–quantum dot composites to polarized and UV light in single-phase microflows. In the range of flow velocities up to 10 mm/s, the flow modes of microfluidic devices were found to correlate with the orientation of liquid crystals, dispersion of quantum dots in homogeneous microflows and the resulting luminescence response of these dynamic systems to UV excitation. We developed a Matlab algorithm and script to quantify this correlation by performing an automated analysis of microscopy images. Such systems may have application potential as optically responsive sensing microdevices with integrated smart nanostructural components, parts of lab-on-a-chip logic circuits, or diagnostic tools for biomedical instruments.

## 1. Introduction

Microfluidic devices and laboratories-on-a-chip broaden horizons for the synthesis and application of photonic particles [1,2,3]. Microfluidic circuits offer additional options for the tailored synthesis of photonic micro- and nanoparticles [4,5,6] and their applications in biosensing [7,8], medical diagnostics [9,10], and fabricating labels with anti-counterfeiting capabilities [11,12,13].

Integrating microfluidic devices with optically active functional nanomaterials such as quantum dots (QD) makes a substantial contribution to advancing applications of fluidic micromachines in nanotechnology [14,15], chemical and bioanalysis [16,17,18], the synthesis of target drug delivery systems [19,20,21] or nanomedicine [22]. Current research activities also focus on fine-tuning the microscale synthesis of quantum dots [23,24,25,26] and conjugating them with soft matter [15,21,27].

Among organized media studied and applied in microfluidic confinement, liquid crystals (LC) are among the most suitable and promising materials for photonics [28,29,30]. Compared to quantum dots at a microscale, integrating liquid crystals with microfluidic chips offers an alternative approach to designing microscale temperature and flow sensors [29,31,32,33] or optically active functional microcapsules [34,35,36]. Confinement offers a simultaneous and programmable control over the optical behavior of LC systems by a number of factors such as flow [37,38,39], temperature [29,33], or light [40].

Doping liquid crystal materials with quantum dots allows to combine their advantages as optically active materials [41,42,43] and offers a synergistic effect to applications of the resulting composites such as displays [44] or security labeling [45]. As opposed to studying and applying such composites in macroscopic conditions, microfluidic research in composites of LC with quantum dots or similar functional nanomaterials is an emerging area represented by mostly pioneering publications, which focus, for example, on composite microdroplets synthesized by microfluidic techniques [45,46]. In this respect, a detailed analysis on the impact of various factors such as flow conditions on optical properties of LC-QD composites in microfluidic channels may bring new insights into fine-tuning their optical responses at the microscale and enhancing the photonic application capabilities of fluidic micromachines.

This work continues our research in nematic LC systems [47] and LC-QD composites [43,48] at the microscale and macroscale. In our previous works, we focused on analyzing the impact of flow conditions in microchannels on the orientation behavior of liquid crystals in single-phase and multiphase microflows and characterized the sensitivity of such systems to variable flows in polarized light.

On the other hand, luminescence is an intrinsic property of quantum dots, which is the cornerstone of their sensing capabilities. This work, therefore, aims specifically at adding the luminescence behavior of quantum dots to the portfolio of possible optical responses of LC-QD composites to variable microfluidic flows. We studied the optical behavior of LC-QD hybrid materials in variable flow conditions in both polarized and UV light in single-phase microflows in straight rectangular microchannels. We analyze a correlation between orientation states of the LC matrix in a microchannel, optical responses of the LC material under polarizers, and the luminescence behavior of quantum dots. The novel contribution of this work is also to offer automated polarized and UV microscopy image analysis capabilities. We offer a Matlab algorithm for image analysis that obtains a quantitative correlation between the optical state of the LC-QD composite and the flow velocity.

## 2. Materials and Methods

### 2.1. Materials

Microfluidic devices were fabricated from polydimethylsiloxane (PDMS) Sylgard^TM^ 184 silicone elastomer. PDMS was purchased from Dow Corning (Midland, MI, USA) and used as received. It came as a two-part elastomer kit (the pre-polymer and curing agent). SU-8 3050 photoresist (Microchem Corp., Westborough, MA, USA) and was used to produce a mold for microfluidic chips.

For the liquid crystal phase, the nematic liquid crystal N-(4-methoxybenzylidene)-4-butylaniline (MBBA) was used. It was purchased from Reachem, Moscow, Russia, and used as received. It exhibits liquid crystal properties at room temperature.

In this work, pre-synthesized core–shell quantum dots were used. In quantum dots, the CdSe core was coated by two shells: the internal CdS shell and the external ZnS shell. The details of synthesis of quantum dots are provided in Supplementary Materials.

The PDMS surface is initially hydrophobic, and its contact angle was high (>100°) according to our contact angle measurement experiments. Before performing microfluidic experiments, microchannel walls were pre-processed by infusing surfactant solutions (sodium dodecyl sulphate—SDS) for 10 min to increase the wettability of internal PDMS walls and favor the orientation of liquid crystal molecules with respect to microchannel walls.

SDS was purchased from BDH Limited, Poole, UK, and used as received. SDS was sold as a powder. Bulk samples of pre-micellar 5 × 10^−3^ mol/L SDS were produced by dissolving dry surfactant in water. At this concentration, SDS is below its critical micellization concentration, which is 8.4 × 10^−3^ mol/L [49]. The addition of surfactant provides a smooth and reproducible formation of uniform droplets in microfluidic confinement [50]. Bidistilled water was used for the aqueous phase. Before performing microfluidic experiments, the solvent was passed through 0.45 µm Millipore polytetrafluoroethylene (PTFE) filters by Merck, Darmstadt, Germany.

### 2.2. Methods

#### 2.2.1. Synthesis of LC-QD Composite and Characterization of Components

Composites of liquid crystal and quantum dots were prepared by doping the LC phase with the dispersion of quantum dots in hexane. The composites were agitated by a magnetic stirrer at 50 °C for 5 h to remove hexane. The concentration of quantum dots in the composite was 0.25 wt %.

The photoluminescence emission spectra of quantum dots and the composite were recorded by a Varian Cary Eclipse spectrofluorimeter (Agilent, Santa Clara, CA, USA). The hydrodynamic diameter of quantum dots was measured by dynamic light scattering (DLS) using a Malvern Zetasizer Nano ZS system (Malvern Panalytical, Malvern, UK).

#### 2.2.2. Recording and Processing Microscopy Images of Microflows

The preliminary characterization of LC and LC-QD flows in microfluidic confinement was performed by digital optical microscopy using a Levenhuk D320 optical microscope (Levenhuk, Tampa, FL, USA). Microscopy images were captured at 100× magnification using a ToupCam E3ISPM08300KPB camera (Touptek, Hangzhou, China).

The orientation behavior of the LC material and LC-QD composites in microfluidic flows were studied by polarized optical microscopy (POM) using an Olympus BX51 microscope (Olympus, Tokyo, Japan) equipped with a high-precision Linkam heating system. Microscopy images were captured at 100× and 500× magnification using a ToupCam E3ISPM08300KPC camera (Touptek, Hangzhou, China).

The luminescent properties of LC-QD composites in microchannels were studied by an Olympus BX43 fluorescent microscope (Olympus, Tokyo, Japan). Microscopy images were captured at 100× magnification using a ToupCam E3ISPM05000KPA camera (Touptek, Hangzhou, China).

Polarized and fluorescent microscopy images were processed by Matlab 2021b software. For processing, microscopy images were taken at identical microscope settings. The luminance components of polarized microscopy images and the red color components of fluorescent microscopy images were extracted and processed by the pre-developed Matlab script.

#### 2.2.3. Fabricating Microfluidic Devices and Preparing Experimental Setup

Microfluidic devices were fabricated using standard photolithography techniques [51]. The chips with rectangular microchannels were produced by this technology. The length, width, and height of all the microchannels were 15 mm, 200 μm, and 100 μm, respectively. SU-8 photoresist and a transparent photomask with a negative image of a microchip were used to produce a 100 µm thick mold of microfluidic chips on top of a 3-inch silicon wafer. PDMS pre-polymer was mixed with a curing agent, poured over the mold, and allowed to cure for 4 h in a 60 °C oven. Once cured, PDMS was peeled off the mold and bonded to a flat PDMS slab via 1 min plasma treatment by Harrick Plasma Cleaner PDC-23G, Ithaca, NY, USA. The PDMS device was then heated in an oven at 180 °C for 1 h to complete the bonding of the two polymer layers.

The LC and aqueous phases were infused into microfluidic devices using Shenchen ISPLab01 syringe pumps (Baoding Shenchen Precision Pump Co. Ltd., Baoding, China), which provide a minimal flow rate of 0.001 µL/min. In this work, the flow velocities of the LC material, LC-QD composite, and the aqueous phase were varied in the range up to 10 mm/s. To provide the same hydraulic paths for fluids to all the inlets, PTFE tubes of identical lengths (10 cm) and internal diameters that fit the same needle tips inserted into microchip outputs (20 G-type needles, 0.9 mm diameter) were used. These tubes were connected to identical 1 mL syringes installed into syringe pumps.

## 3. Results

### 3.1. Preliminary Characterization of LC and LC-QD Composites in Microfluidic Confinement

Microfluidic devices and the confined environment of microchannels often require specific conditions for infusing and processing both organic and aqueous fluids. The viscosity, temperature behavior and optical characteristics of liquid crystal and quantum dot systems are important factors for designing applicable microfluidic devices and ensuring the compatibility of media with microfluidic experiments.

**Figure 1:**
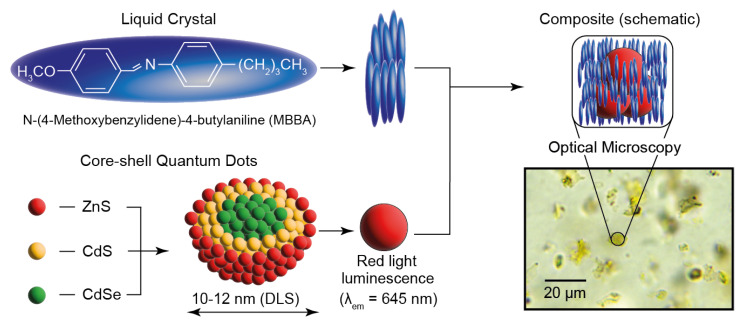
Components, schematic microstructure and optical microscopy images of MBBA composites with CdSe-CdS-ZnS core–shell quantum dots.

At the first stage of this work, we performed a preliminary analysis of components of the LC-QD composite and its compatibility with microchannel confinement. Figure 1 shows a schematic representation of LC molecules, quantum dots, and their composites and the results of their characterization by applicable spectroscopy and microscopy methods.

According to the synthesis procedure, the core–shell quantum dots used in this work consist of the internal CdSe core that is covered with CdS and ZnS shells. According to dynamic light scattering experiments, the average hydrodynamic diameter of quantum dots dispersed in hexane is about 10–12 nm. As compared with smaller size QD particles, the emission maximum of such quantum dots is supposed to be in a longer-wave visible range [43]. It agrees with spectrofluorimetry studies, which report that the emission peak of these quantum dots is in the red light range both individually and in composite (λ_em_ ≈ 650 nm). This wavelength is within the transmittance range of PDMS [52], so we can expect a smooth detection of QD emission in microflows.

MBBA is an intensively studied liquid crystal material, which attracts a sustainable research interest both individually and as a matrix for various composites [53,54]. It exhibits nematic mesophase at room temperature (its clearing point is about 38 °C [54]). At room temperature, MBBA is represented by a yellow turbid liquid, which could be easily infused into standard PDMS microchips, according to our preliminary microfluidic tests. Such microchips can operate at room temperature and require no internal or external heating system to obtain a nematic MBBA mesophase.

The optical microscopy photo in Figure 1 shows an image of the LC-QD composite at 500× magnification. We can see that the structure of the composite is non-homogeneous and includes clusters of quantum dots in the size range of approximately 5–30 μm. The size of such QD clusters is much smaller than the standard width of microfluidic channels (~100–200 μm), so the composites can be smoothly infused into conventional microfluidic devices.

**Figure 2:**
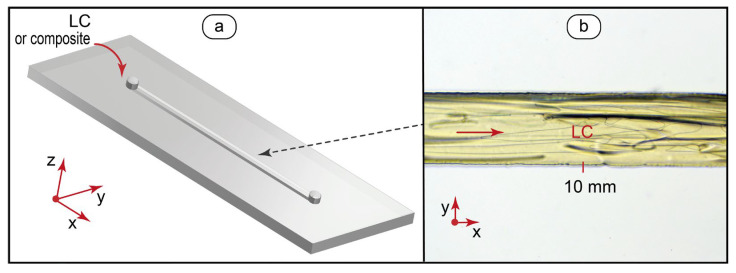
Design of microfluidic chips and flow testing: (**a**) microfluidic device with a straight microchannel for generating single-phase LC and LC-QD microflows; (**b**) bright field optical microscopy image of a single-phase flow of the pure MBBA liquid crystal at 0.5 mm/s.

Therefore, we designed and fabricated microfluidic devices for MBBA-QD composites with the channel widths of 200 µm and heights of ≈100 µm, so they could smoothly incorporate such clusters of quantum dots. Figure 2 demonstrates the designs of the respective microfluidic devices and test microscopy photos of single-phase and two-phase microflows.

For single-phase experiments (Figure 2a), we fabricated single-channel microfluidic chips with a channel width of 200 μm and length of 15 mm. Such microchips allowed us to generate a smooth flow of the LC phase (Figure 2b) or a composite in the studied flow velocity range (up to 10 mm/s) and above.

Thus, MBBA-QD composites are represented by microscale QD clusters in an LC matrix. Such disperse systems are compatible with microfluidic confinement and can be processed by standard microfluidic devices. The optical properties of the LC phase and QD emission can be studied by applicable microscopy methods. The next stage of this work focused on characterizing orientation behavior of the LC phase by polarized optical microscopy and luminescence of quantum dots by fluorescence microscopy.

### 3.2. Optical Behavior of LC-QD Microflows in Polarized and UV Light

#### 3.2.1. Orientation Behavior of Confined MBBA and MBBA-QD Composite

Nematic liquid crystal flows demonstrate a flow-dependent orientation behavior in microfluidic confinement [37]. Depending on the orientation of LC molecules with respect to the view direction, a variety of optical responses are generated by microchannels with a mobile mesophase in polarized light: from a homeotropic orientation and a planar orientation of a continuous LC phase to characteristic cross-shaped textures of LC microdroplets [28,39].

A useful approach to quantify orientation of the LC director such as its tilt angle is comparing their interference colors with a Michel–Levy chart [55,56,57,58]. The authors in [56] evaluated the in-plane orientation of the liquid crystal by rotating the LC cell between crossed polarizers and observing changes in interference colors. In [59], the rotation of polarizers to 45° was used to confirm the orientation of LC specifically in microchannel confinement.

**Figure 3:**
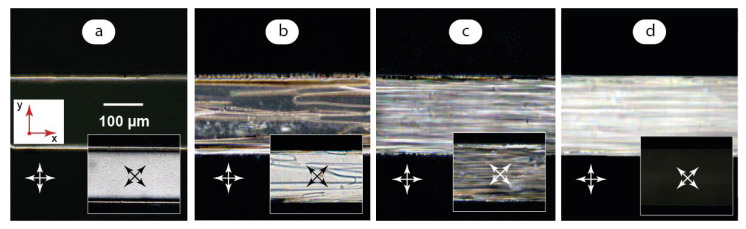
Polarized microscopy images of LC microflows: (**a**) U → 0; (**b**) U = 0.5 mm/s; (**c**) U = 1 mm/s; (**d**) U = 10 mm/s. Red arrows demonstrate coordinate axes with respect to Figure 2. Crossed arrows indicate positions of polarizers.

To characterize the orientation behavior of the LC matrix in a LC-QD composite in single-phase flows, we performed polarized optical microscopy studies of LC microflows at flow velocities up to 10 mm/s. The results are summarized in Figure 3.

Figure 3a was obtained by infusing LC to the microchannel first and then turning off the syringe pump to obtain an immobile LC phase. At flows below approximately 0.1 mm/s, the microchannel environment is represented by a nearly uniform dark field at crossed polarizers except for ~10 μm bright stripes at microchannel boundaries. Such an optical response was reported in [37,39]. We also observed it in our previous studies of similar nematic LC flows [47]. Such an image is supposed to represent a homeotropic orientation of the LC phase with the domains aligned perpendicular to microchannel walls and the view direction.

The POM image with polarizers rotated by 45° (Figure 3a inset) shows, however, a bright field image in such conditions. No visible differences in color are observed along the microchannel, which could indicate no differences in LC alignment to be distinguished by the Michel–Levy chart. Such an alignment was reported to be more typical for a uniform director orientation in a position tilted to the flow axis [37] or aligned along it [59].

It should be noted that the alignment state shown in Figure 3a was stable only in weak (<0.1 mm/s) flow conditions. The immobile LC phase demonstrated sequential transitions from uniform bright to dark colors of different brightness at crossed polarizers both in 0° and 45° positions within 30 min observation after the flow was fully stopped. The coincidence of dark fields at both positions of crossed polarizers did take place, but it was very unstable.

Such a behavior can be associated with the fact that achieving a stable homeotropic alignment requires processing microchannels with aligning reagents [37]. Although microchannels were pre-processed with surfactant (1 mmol SDS) before LC experiments, added surfactant turned out to be insufficient to provide a stable homeotropic orientation. In their turn, weak flows may perform as a stabilizing orientation factor for LC molecules that favors their alignment with respect to the microchannel axis. It should be emphasized that the director orientation stayed uniform along the microchannel in the observed zero flow or weak flow conditions: no separate zones of different colors or light intensities were detected along the microchannel.

At flow velocities higher than 0.2 mm/s, non-uniform stripes appear in the LC flow under polarized light (Figure 3b). Polarizers rotated to 45° show (Figure 3b inset) show a residual bright field with the color close to that demonstrated in the Figure 3a inset. It indicates that LC molecules still tend to align uniformly with respect to the flow axis in faster flow conditions.

A further increase of the flow velocity up to 1 mm/s makes this non-uniform structure predominant (Figure 3c). The rotation of polarizers (Figure 3c inset) provides a similar irregular pattern with a variety of light intensities and colors. If we apply the Michel–Levy color chart approach to Figure 3c, we can conclude that it demonstrates a rather irregular alignment of LC domains with arbitrary angles with respect to the microchannel axis, although a certain ordering effect of flow on the stripes is observed.

At higher flow velocities, LC domains start to align along the flow direction. At the flow velocity above ~5 mm/s, the orientation of LC domains is predominantly planar (Figure 3d) and shows a characteristic bright field in polarized light with crossed polarizers at 0° and a darker field at 45°.

**Figure 4:**
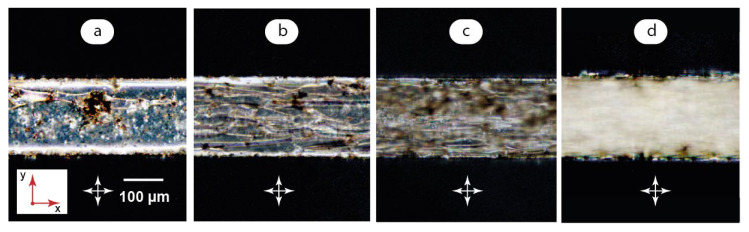
Polarized microscopy images of LC-QD microflows: (**a**) U → 0; (**b**) U = 0.5 mm/s; (**c**) U = 1 mm/s; (**d**) U = 10 mm/s. Red arrows demonstrate coordinate axes with respect to Figure 2. Crossed arrows indicate the positions of polarizers.

At the next stage of this work, we studied the impact of added quantum dots on the behavior of the LC matrix under polarized light. Figure 4 summarizes the results obtained in the same flow conditions as those demonstrated in Figure 3.

The impact of quantum dots on the optical state of the composite at U→0 is shown in Figure 4a. We can see a mostly uniform alignment of the LC matrix in slow flow or zero flow conditions. Quantum dot aggregates, however, also perform as aligning additives for the LC molecules and show bright spots around QD clusters in polarized light. The rotation of polarizers changes the colors of the microchannel medium in a way similar to that shown in Figure 3a for a pure LC matrix.

QD aggregates were found to be unstable in microchannel flows. An increase of flow velocities above 0.5 mm/s results in the decomposition of QD aggregates to smaller clusters (Figure 4b,c). Increasing shear stresses in microscale flows favors the homogenization of the LC-QD composite that agrees with our previous studies of a similar hybrid system [48]. At the same time, QD aggregates favor the formation of instabilities in the LC matrix: a chaotic pattern in Figure 4b is more pronounced as compared to that of a pure LC matrix in the same flow conditions (Figure 3b). Transition to an irregular pattern occurs in the 0.5–1 mm/s range, which is approximately 0.3 mm/s lower than in a pure LC matrix: the composites in Figure 4b,c show much more similarities than a pure LC matric at the same flow velocities in Figure 3b,c.

Finally, a uniform bright field is achieved in fast flows approaching 10 mm/s (Figure 4d) similar to that demonstrated by a pure LC matrix in Figure 3d upon its transition to a predominant orientation along the flow.

**Figure 5:**
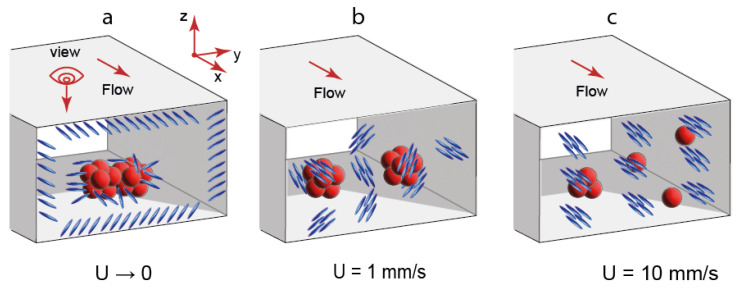
Suggested orientation of LC molecules and state of QD aggregates in LC-QD composites at various flow velocities: (**a**) U = 0.1 mm/s; (**b**) U = 1 mm/s; (**c**) U = 10 mm/s.

A suggested confined orientation of LC molecules in the presence of QD aggregates is shown in Figure 5. In slow flow conditions (Figure 5a), the orientation is predominantly uniform. In slow flows, LC molecules are supposed to align in a certain direction with respect to the microchannel axis. QD aggregates coexist with the LC phase and also perform as additional directors for its molecules. It should be noted that an alignment of LC molecules in zero flow conditions was found to be unstable but still uniform.

In microflows at 1 mm/s (Figure 5b), a uniform orientation of LC molecules is replaced with an irregular pattern of LC domains. The size of QD aggregates decreases in faster flows. Finally, LC domains tend to align along the flow axis (Figure 5c). However, such an alignment is not perfect, and residual inhomogeneities can be still detected in Figure 3d and Figure 4d.

The Reynolds number is a key parameter that characterizes flow transitions in single-phase microfluidic systems, so it is interesting to evaluate it for the conditions shown in Figure 3 and Figure 4. For aqueous microflows, for example, this parameter is generally small and corresponds to a laminar flow mode, which was observed in our previous experiments [60]. The Reynolds number depends on the fluid viscosity, and liquid crystals demonstrate non-uniform flow-dependent viscosities. As opposed to aqueous flows, the Reynolds number is expected to provide only an approximate characterization of LC flows at various flow velocities and non-uniform viscosities resulting from changes in the alignment of LC molecules in variable flows. The data in [61] allow us to evaluate MBBA viscosities according to Leslie–Ericksen theory for various Miesowicz geometries of LC molecules aligned parallel or orthogonal to the velocity vector. According to Figure 3, MBBA may exhibit intermediate orientation states as compared to these geometries in our microfluidic experiments. We can consider, therefore, the viscosities found for these geometries as the boundaries of a possible MBBA viscosity range in our experiments and evaluate the resulting Reynolds number. All the viscosity coefficients reported in [61] provided Re < 1 for the microchannel width of 200 µm and velocities up to 10 mm/s. The LC flow transitions shown in Figure 3 occur, therefore, at low Reynolds numbers.

It should be noted that microfluidic devices remained stable and operational at increasing flow velocities. Although flow velocities of several millimeters per second are quite high for microfluidic experiments performed in microscale width channels, we observed no deformation of such a microchannel at LC flow rates up to 20 µm/s. A possible effect of flow pressure can be expected for long channels of serpentine microchips with much larger hydraulic resistance. The major flow-induced effects of LC alignment and QD luminescence were observed, however, at flow velocities below 1 mm/s. Thus, such operation modes can be potentially applicable to a broader range of microchip designs.

The aggregates of quantum dots were large at zero flow conditions. Their aspect ratio (aggregate diameter to microchannel height) was greater than 0.3 in zero flow conditions or flows slower than 0.2 mm/s. The aggregates, however, did not exert a negative impact on microchannel environment. They turned out to be easily deformable inside a microchannel and were therefore supposed to put virtually no pressure on microchannel walls. The aggregates were also sensitive to flow: their size reduced to approximately 20–30 μm upon an applied flow of 0.3–0.5 mm/s and less in faster flows.

Thus, MBBA-QD composites demonstrate a flow-dependent orientation behavior of the LC matrix and aggregative the behavior of quantum dots. It resulted in a variety of optical responses to polarized light. At the next stage of this work, we analyzed an impact of flow-dependent properties of the composite on the luminescence properties of quantum dots in UV light.

#### 3.2.2. Luminescence Behavior of MBBA-QD Composite Microflows

**Figure 6:**
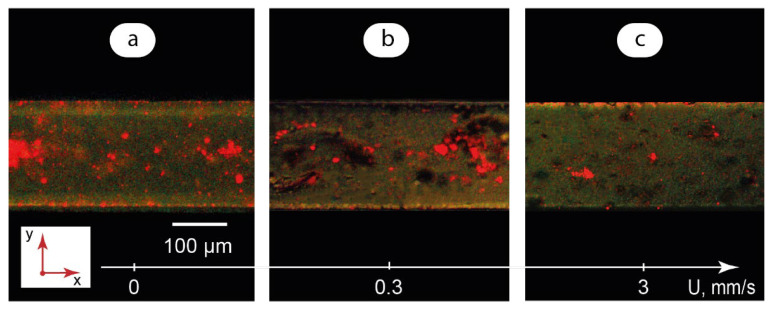
Fluorescence microscopy images of single-phase LC-QD composite microflows: (**a**) U → 0; (**b**) U = 0.3 mm/s; (**c**) U = 3 mm/s.

Fluorescence microscopy is a key method to unleash the sensing potential of lab-on-a-chip, especially in cutting-edge biomedical applications [17,62,63] and organ-on-a-chip platforms [64]. Therefore, analyzing the luminescence behavior of LC-QD hybrids in microfluidic confinement may reveal new approaches to sensing applications of such composites in lab-on-a-chip devices. At this stage of the work, we studied the impact of flow velocity on the luminescence behavior of the composite. Figure 6 summarizes the results of the respective fluorescence microscopy experiments.

Excitation by UV light initiates a red-light emission of quantum dots in a confined immobile composite (Figure 6a). Some of the QD clusters are absorbed on microchannel walls and represent bright red light spots on the microscopy image. The largest clusters are about 50–60 μm in diameter. The height of the microchannel is 100 μm, so all the QD clusters can be potentially fully immersed in the LC matrix. The luminescence from such clusters is easily detected in Figure 6a as turbid red spots throughout the microchannel.

In the flow of the composite at 0.3 mm/s (Figure 6b), large clusters of quantum dots still exist. They exhibit, however, a much weaker luminescence than in immobile composite. The clusters are mostly represented by dark spots with limited red color areas. The major contribution to luminescence is provided by smaller QD clusters.

Finally, Figure 6c shows a residual red light emission from small QD aggregates at 3 mm/s. The majority of QD aggregates are represented by turbid dark spots inside the LC matrix.

The luminescence behavior of the composite demonstrated in Figure 6 correlates with the orientation states of the LC matrix (Figure 3). The most intensive luminescence was observed for a uniformly aligned LC matrix in zero or slow flow conditions. The transition from a uniformly aligned state to an irregular pattern of domains reduced QD emission.

A possible reason for such an optical behavior of quantum dots is the Rayleigh scattering of electromagnetic radiation and anisotropy of the LC matrix. In slow flows of microfluidic LC-QD composites, the molecules of the LC matrix are supposed to be aligned uniformly with respect to microchannel walls. Such an orientation may initiate a less intensive scattering of UV light on its path to the QD cluster inside a microchannel as compared to faster flows with an irregular pattern of LC domains.

In addition, the scattering intensity is inversely proportional to λ^4^, according to the Rayleigh scattering law, where λ is the wavelength of light. Excitation UV light (λ = 365 nm), therefore, is scattered more intensively than emission light from QD clusters (λ = 645 nm) with the factor of (645/365)^4^ ≈ 10. In LC-QD flows, UV light intensity can be insufficient to excite a substantial emission from QD clusters inside a microchannel. Such clusters are represented by dark spots in Figure 6b,c. The major contribution to luminescence is made by small QD clusters absorbed on microchannel walls or segments of large QD clusters near microchannel walls, where a thickness of the LC matrix and the resulting scattering are minimal.

Finally, the decomposition of aggregates and reduction in their adsorption to microchannel walls favors their uniform distribution inside the LC matrix and reduces a contact with microchannel walls in fast flows that should also contribute to minimizing the emission from the composite (Figure 6c).

#### 3.2.3. Quantifying the Impact of Flow Conditions on Optical Responses of Confined LC-QD Composite

The automated analysis of microscopy data with image processing software offers a convenient tool to characterize the optical behavior of microfluidic flows. To reveal possible quantitative correlations between microflow conditions and optical responses of the LC-QD composite in polarized and UV light, we performed a microscopy image analysis with a pre-developed Matlab script. The details of the image processing algorithm and the respective Matlab script are provided in the Supplementary Materials.

To perform this analysis, a series of LC and LC-QD composite microscopy images were taken in polarized and UV light in addition to the images shown in Figure 3, Figure 4 and Figure 6. All the images in each series were taken with identical microscope settings. These images are shown in Figure S1 in Supplementary Materials.

**Figure 7:**
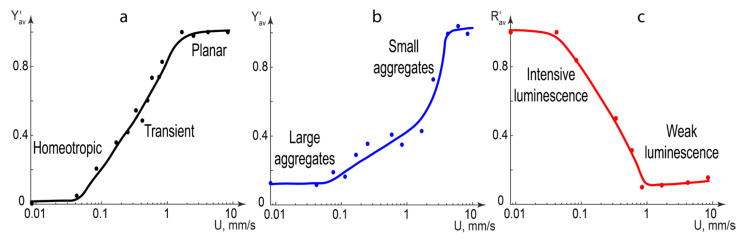
Optical characteristics of microfluidic channels with infused LC or LC-QD composites obtained from polarized and fluorescence microscopy images processed with Matlab: (**a**) reduced average luminance of the pure LC phase in polarized light; (**b**) reduced average luminance of the LC-QD composite in polarized light; (**c**) reduced average red color brightness of the LC-QD composite in UV light.

For polarized microscopy images, their average luminance $\bar{Y}$ of the YCbCr color space [65] was calculated by the Matlab script. For fluorescence microscopy images, their average red color brightness $\bar{R}$ from the RGB data was calculated by the Matlab script. Figure 7 demonstrates the results.

The following reference values were used for image processing. The average luminance ${\bar{Y}}_{0}$ of the pure immobile LC phase was considered to be zero. The average luminance of the pure LC phase at 10 mm/s ${\bar{Y}}_{\max}$ was considered to be maximum. The average red color brightness of the pure LC phase at 10 mm/s ${\bar{R}}_{0}$ was considered to be zero. The average red color brightness of the immobile LC-QD composite at 10 mm/s ${\bar{R}}_{\max}$ was considered to be maximum.

The average reduced luminance (Figure 7a,b) was calculated by the following equation:

(1) $$Y^{\prime }=\frac{\;\bar{Y}-{\;\bar{Y}}_{0}}{{\;\bar{Y}}_{\max}-{\;\bar{Y}}_{0}}$$

The average reduced red color brightness (Figure 6c) was calculated by the following equation:

(2) $$R^{\prime }=\frac{\;\bar{R}-{\;\bar{R}}_{0}}{{\;\bar{R}}_{\max}-{\;\bar{R}}_{0}}$$

Both Y′ and R′ varied, therefore, in the [0;1] range in all the images.

Figure 7a shows the optical states of pure LC phase microflows. The zero luminance zone at the 0° position of crossed polarizers corresponds to a uniform orientation of LC molecules in microflows below 0.1 mm/s. It transforms into the maximum luminance at velocities above 2–3 mm/s through a transient zone where the Y′ changes nearly linearly with logarithmic flow velocity upon a growing contribution of irregular LC patterns.

In Figure 7b, the orientation of LC molecules around QD aggregates in composites increases the initial luminance at flow velocities below 0.1 mm/s. Large and dark QD aggregates decrease the growth of luminance in the 0.1–1 mm/s range as compared with pure LC phase flows. The decomposition of aggregates in faster flows quickly increases the luminance up to its maximum value.

Figure 7c shows that the red color brightness is maximum in flows slower than 0.1 mm/s, which corresponds to undisturbed red light emission from quantum dots in the uniformly aligned LC matrix. In faster flows, the red color intensity decrease is close to linear with logarithmic flow velocity. In flows faster than 1 mm/s, irregular LC patterns are supposed to be responsible for intensive scattering of the excitation UV light. The brightness reaches its minimum corresponding to a residual luminescence from QD clusters absorbed on the microchannel walls.

Thus, processing polarized and fluorescence microscopy images allowed us to quantify the optical response of the LC-QD composite to variable microchannel flow velocity and correlate it with orientation state of the QD matrix, flow behavior of QD aggregates and their luminescence properties.

## 4. Discussion

Composites of MBBA with core–shell CdSe quantum dots were found to generate a variety of optical responses in mobile microchannel confinement both in polarized and UV light. Such an optical behavior of the composite may be attributed to changes in the orientation of domains in the LC matrix. It can be, therefore, conveniently controlled by a single microfluidic parameter (flow velocity up to 10 mm/s).

A quantitative correlation between the flow velocity and average brightness of LC-QD hybrids in polarized and UV light was detected in the flow range up to 2–3 mm/s, which is a typical operation mode of microfluidic devices. Such hybrids can be, therefore, used as luminescence flow detectors or quantitative flow sensors in this range.

At low (below 0.05 mm/s) and high (over 1–2 mm/s) flow velocities, there are two opposite optical states by both the LC matrix in polarized light and QD luminescence in UV. In polarized light, the LC matrix shows a dark field microchannel environment in slow flows and a bright field image in fast flows. Quantum dots demonstrate bright luminescence in UV light in zero or slow flows, which decreases to almost zero in fast flow conditions. Both these effects can be suitable for microfluidic logic applications and setting “0” or “1” logic states and designing flow-controlled optofluidic logic gates. Such logic states can be detected and processed by a simple automated microscopy image analysis with a respective algorithm and used for automated sensing application in lab-on-a-chip tools.

Despite their application potential, the revealed optical effects, however, represent fundamental research results. Studying effects of additional factors on optical properties of confined LC-QD hybrids may contribute to clarifying their potential as nanostructural components of optofluidic devices. The future research activities will focus on studying the impact of temperature and chemical agents (such as surfactants at various concentrations) on optical properties of the studied LC-QD hybrids. Special attention will be given to analyzing the flow and temperature behavior of multiphase systems with LC-QD hybrids confined in microdroplets suitable for further applications as functional photonic microparticles. In this respect, hydrodynamic trapping is a promising approach that allows us to perform experiments with colloid particles and anisotropic systems in microfluidic confinement with a precise control of these parameters [66,67]. Available microfluidic chip designs will allow us to create Stokes traps for LC-QD hybrids and control their behavior with flowrates and temperature as well as additional parameters such as magnetic field.

## 5. Conclusions

MBBA liquid crystal composites with CdSe core–shell quantum dots were found to reversibly generate opposite optical responses in both polarized and UV light in a typical microflow velocity range up to 10 mm/s. In flows slower than 0.05 mm/s, the composite is dark under crossed polarizers at the 0° position and shows intensive red light emission in UV light. These effects change to a bright field in polarized light and almost zero emission in UV light in flows faster than 1–2 mm/s. The change of brightness upon transition between these two states occurs almost linearly with the logarithmic flow velocity. Such a behavior is considered to correlate with a transition of the LC matrix from a uniform orientation at zero or slow flows to an irregular pattern of domains, which then gradually align along the flow axis.

Such optofluidic microdevices offer new options for applications in photonics as luminescent flow sensors or lab-on-a-chip binary logic circuits as well as optically active tools for medical diagnostics.

## Data Availability

Not applicable.

## Supplementary Materials

The following supporting information can be downloaded at: https://www.mdpi.com/article/10.3390/mi14050990/s1, Figure S1. Processed microfluidic images of MBBA flows in microfluidic channel at various flow velocities [68,69].
