# Supplementary material for: Dynamic Flow Control over Optical Properties of Liquid Crystal–Quantum Dot Hybrids in Microfluidic Devices
Source: Micromachines (Basel). 2023 Apr 30;14(5):990. doi: 10.3390/mi14050990 (PMC10222004; doi:10.3390/mi14050990)
Supplement: Supplementary file 1 [file micromachines-14-00990-s001.zip › micromachines-2354204-supplementary.pdf]

Image Processing Algorithm

For image processing, a series of polarized and fluorescence microscopy images was taken in the 0-10 μl/min flowrate range. The flowrates were transformed into respective flow velocities. All the images were taken at identical microscope and image capturing software settings. For processing polarized microscopy images, we extracted the “Y” (luminance) component of the YCbCr color space of tiff image files [1].

The extracted “Y” component of images is represented by a matrix (row-column):

$$\mathrm{Y} = \begin{pmatrix} \mathrm{Y}_{11} & \mathrm{Y}_{12} & \cdots & \mathrm{Y}_{1j} \\ \mathrm{Y}_{21} & \mathrm{Y}_{22} & \cdots & \mathrm{Y}_{2j} \\ \cdots & \cdots & \cdots & \cdots \\ \mathrm{Y}_{i1} & \mathrm{Y}_{i2} & \cdots & \mathrm{Y}_{ij} \end{pmatrix} \quad (1)$$

where i and j are the width and height of the image, respectively.

The next step is to calculate the average luminance $\overline{\mathrm{Y}}$ of all pixels in the image:

$$\overline{\mathrm{Y}} = \mu(\mathrm{Y}) \quad (2)$$

For the algorithm, we calculate two reference mean values of Y.

For all the polarized microscopy images (pure LC phase or the LC-QD composite series), the first reference value $\overline{\mathrm{Y}}_0$ is the mean luminance of the pure liquid crystal phase at zero flowrate. The second reference value $\overline{\mathrm{Y}}_{\mathrm{max}}$ is the mean luminance of the last image in a series that corresponds to the maximum flowrate.

The first image of the pure LC phase in homeotropic orientation is not completely dark ($\overline{\mathrm{Y}}_0 \approx$ 50-70) with the color space maximum of 256. It is convenient, however, to assume $\overline{\mathrm{Y}}_0$ as the zero point and introduce zero-point correction for all the images:

$$\overline{\mathrm{Y}}' = \overline{\mathrm{Y}} - \overline{\mathrm{Y}}_0 \quad (3)$$

and for the second reference image:

$$\overline{\mathrm{Y}}'_{\mathrm{max}} = \overline{\mathrm{Y}}_{\mathrm{max}} - \overline{\mathrm{Y}}_0 \quad (4)$$

To facilitate comparative analysis of image processing results, the Matlab script is designed to calculate reduced average luminance values in the [0;1] range. The script calculates reduced mean luminance values of all images by using the corrected luminance of the image with the maximum flowrate. The reduced mean luminance of a polarized microscopy image is, therefore, calculated as follows:

$$\mathrm{Y}' = \frac{\overline{\mathrm{Y}} - \overline{\mathrm{Y}}_0}{\overline{\mathrm{Y}}_{\mathrm{max}} - \overline{\mathrm{Y}}_0} \quad (5)$$

We can see in Eq. 5 that the reduced average luminance of the first and last images in the LC image row will be equal to zero and one, respectively. All the other images will take intermediate values of Y' in the [0;1] range.

For processing fluorescence microscopy images, we extracted the “R” (red color) component of the RGB matrix of the respective image files.

The extracted “R” component of images is represented by a matrix (row-column):

$$\mathrm{R} = \begin{pmatrix} \mathrm{R}_{11} & \mathrm{R}_{12} & \cdots & \mathrm{R}_{1j} \\ \mathrm{R}_{21} & \mathrm{R}_{22} & \cdots & \mathrm{R}_{2j} \\ \cdots & \cdots & \cdots & \cdots \\ \mathrm{R}_{i1} & \mathrm{R}_{i2} & \cdots & \mathrm{R}_{ij} \end{pmatrix} \quad (6)$$

where i and j are the width and height of the image, respectively.

The next step is to calculate the average red color intensity $\overline{\mathrm{R}}$ of all pixels in the image:

$$\overline{\mathrm{R}} = \mu(\mathrm{R}) \quad (7)$$

For the algorithm, we calculate two reference mean values of R.

For all the fluorescence microscopy images (the LC-QD composite series), the first reference value $\overline{\mathrm{R}}_{\mathrm{max}}$ is the mean red color intensity of the LC-QD composite at zero flowrate. The second reference value $\overline{\mathrm{R}}_0$ is the mean red color intensity of the pure LC phase at the maximum flowrate.

It is convenient to assume $\overline{\mathrm{R}}_0$ as the zero point and introduce zero-point correction for all the images:

$$\overline{\mathrm{R}}' = \overline{\mathrm{R}} - \overline{\mathrm{R}}_0 \quad (8)$$

and for the first reference image:

$$\overline{\mathrm{R}}'_{\mathrm{max}} = \overline{\mathrm{R}}_{\mathrm{max}} - \overline{\mathrm{R}}_0 \quad (9)$$

To facilitate comparative analysis of image processing results, the Matlab script is designed to calculate reduced average red color intensity values in the [0;1] range. The script calculates reduced mean red color intensity values of all images by using the corrected luminance of the image with the maximum flowrate. The reduced mean luminance of a polarized microscopy image is, therefore, calculated as follows:

$$\mathrm{R}' = \frac{\overline{\mathrm{R}} - \overline{\mathrm{R}}_0}{\overline{\mathrm{R}}_{\mathrm{max}} - \overline{\mathrm{R}}_0} \quad (10)$$

We can see in Eq. 10 that the reduced average luminance of the first image in the LC-QD composite image row will be equal to one. The last image will demonstrate the residual contribution to the red color intensity from quantum dot traces adsorbed on microchannel walls. All the other images will take intermediate values of R'.

Synthesis of Quantum Dots

Core-shell CdSe/CdS/ZnS quantum dots were synthesized by a colloidal method described in [2].

Image Processing Matlab Script

```
%Microscopy Image Analysis Script
%Default microscopy method: polarized microscopy

%Select image location folder:
% a = uigetdir;
%Select image data file name:
% b = uigetfile;
% Process image data file into Matlab array:
T = readtable(strcat(a,'\',b));
A = table2array(T);
FlowRate = A(:,1);
FileName = A(:,2);

%Calculate the flow velocity U from the flowrate values and microchannel
width and height:
W = 200; H = 100;
U=FlowRate/W/H*10^6/60;

%Select reference files (the first file at zero flowrate and the last file
%at maximum flowrate):
c_in = num2str(FileName(1));
c_f = num2str(FileName(end));
d = '.tif';
t_in = Tiff(strcat(a,'\',c_in,d));
t_f = Tiff(strcat(a,'\',c_f,d));
%Change for fluorescence microscopy:
%c_in = num2str(FileName(end));
%c_f = num2str(FileName(1));
%d = '.tif';
%rgb_in = imread(strcat(a,'\',c_in,d));
%rgb_f = imread(strcat(a,'\',c_f,d));

 %Process reference image files to extract pixel-by pixel brightness:
imageData_in = read(t_in);
imageData_f = read(t_f);
array_in = imageData_in(:,:,1);
array_f = imageData_f(:,:,1);
%Change for fluorescence microscopy:
%array_in = rgb_in(:,:,1);
%array_f = rgb_f(:,:,1);

 %Calculate average brightness of the image at zero flowrate:
m = mean(array_in,'all');
 %Adapt the brightness of the initial image to set its brightness equal to
zero:
array_in1 = array_in-m;

%Adapt the brightness of the image at maximum flowrate:
array_f1 = array_f-m;

%Calculate average reduced brightness of the image at maximum flowrate in the
[0-1] range:
preval = mean(array_f1,'all')/256;

%Set the correction coefficient to consider the brightness of the last
image equal to 1:
corr = 1/preval;

%Correction coefficient 'corr' will be used in further calculations to
%adapt all the other images to brightness of the reference (last) image
```

```
%Set the loop to consecutively read all the images in the folder:
h = numel(FileName);
bright = zeros(h,1);

for k = 1:h
c = num2str(FileName(k));
t = Tiff(strcat(a,'\',c,d));
imageData = read(t);
array = imageData(:,:,1);
array_1 = array-m;
val = mean(array_1,'all')/256*corr;

%Add the average reduced brightness of the figure at specific flowrate to the
results:
bright(k) = val;
 %Plot the images that show the brightness of their pixels:
 column = 3;
row = fix(h/column) +1;
 y_array = numel(array_1(:,1));
x_array = numel(array_1(1,:));
y_surf = linspace(1,200,y_array);
x_surf = linspace(1,200,x_array);
[X,Y] = meshgrid(x_surf,y_surf);
 intens = im2double(array_1)*corr/2;
subplot(row, column,k)
 surf(X,Y,intens)
 view(0.3,90)
 axis off
 shading interp
 colormap gray
 colorbar
 c = num2str(FileName(k));
 title(strcat('Flowrate =',c,' µl/min'))
 hold on
 end

%Plot the figure that shows the dependence of the average reduced brightness
of the image on the flowrate:
figure
p = semilogx(U,bright,'o');
p.MarkerFaceColor = 'k';
p.MarkerEdgeColor = 'none';
p.MarkerSize = 6;
axis([U(1) 10 0 1.1*max(bright)])
```

Processed Image Series

Examples of processed polarized microscopy images of the LC flows are shown in Fig. SI1. Same processing operations were performed for images of LC-QD flows in polarized light and LC-QD flows in UV light.

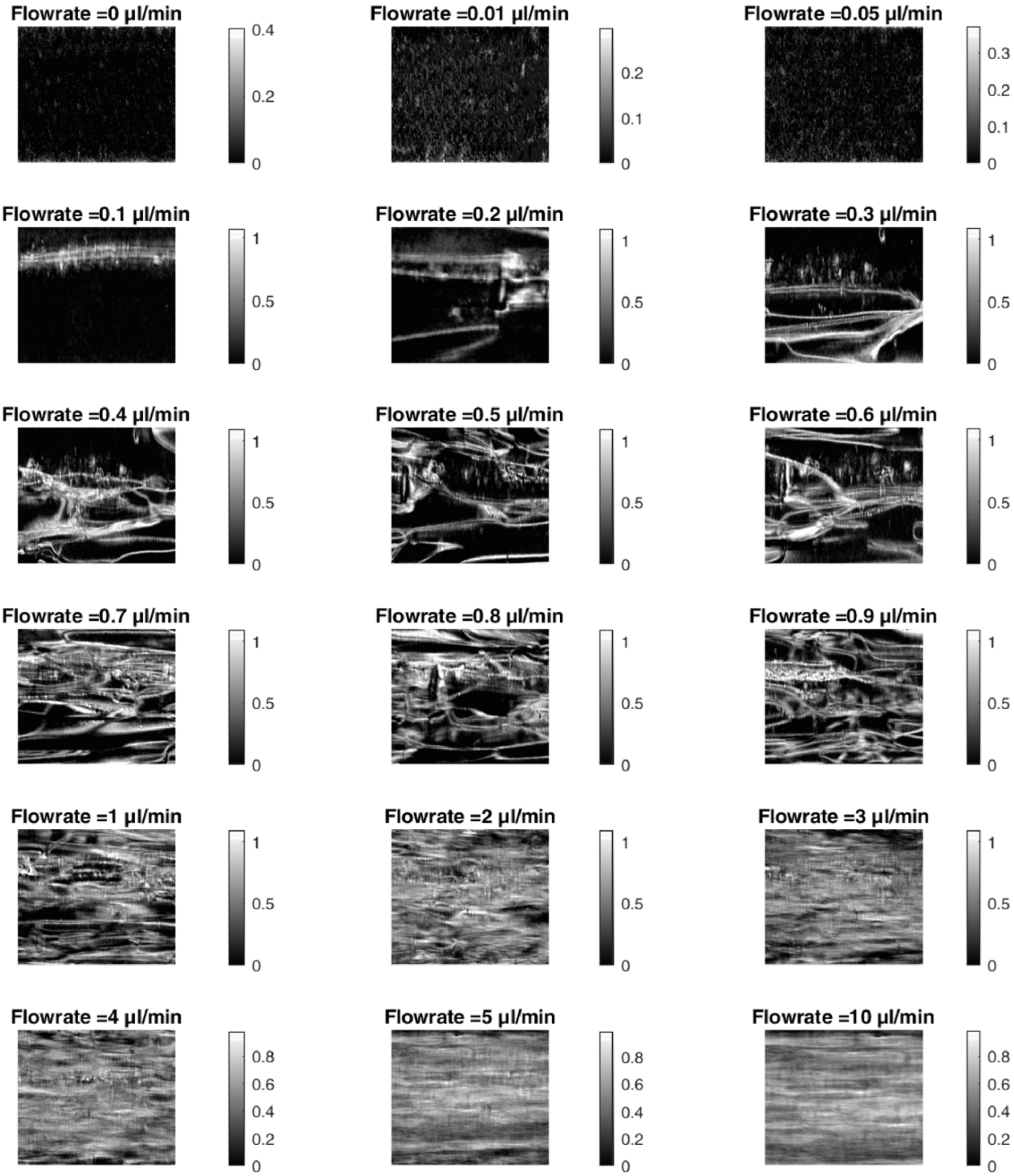

Figure S1. Processed microfluidic images of MBBA flows in microfluidic channel at various flow velocities. Colorbars represent Y' values calculated using the Matlab script.

References

1. Qidwai, U.; Chen, C. H., *Digital image processing: an algorithmic approach with MATLAB*. CRC Press: **2009**; p 294.

2. Galyametdinov, Y. G.; Krupin, A. S.; Sagdeev, D. O.; Karyakin, M. E.; Shamilov, R. R.; Knyazev, A. A., Luminescent Composites Based on Liquid Crystalline Europium({III}) Complex and {CdSe}/{CdS}/{ZnS} Quantum Dots. *Liquid Crystals and their Application* **2022,** *22* (1), 27-38.
